# Robot-assisted radiofrequency ablation versus open resection for osteoid osteoma: an analysis of perioperative outcomes and a rare case of malignant transformation

**DOI:** 10.3389/fonc.2026.1786525

**Published:** 2026-04-21

**Authors:** Kuncheng Wu, Mingxian Xu, Guodong Zhong, Jixiang Shi, Xianbiao Xie, Jingnan Shen, Changye Zou, Jian Tu

**Affiliations:** 1 Department of Musculoskeletal Oncology, The First Affiliated Hospital of Sun Yat-sen University, Guangzhou, Guangdong, China; 2 Zhongshan School of Medicine, Sun Yat-sen University, Guangzhou, Guangdong, China; 3 Guangdong Provincial Key Laboratory of Orthopedics and Traumatology, The First Affiliated Hospital of Sun Yat-Sen University, Guangzhou, Guangdong, China

**Keywords:** malignant transformation, open surgery, osteoid osteoma, perioperative outcomes, robot-assisted radiofrequency ablation

## Abstract

**Introduction:**

Osteoid osteoma (OO) is a rare benign bone tumor predominantly affecting young individuals, with malignant transformation reported only in exceptional cases. This study aimed to compare clinical outcomes between robot-assisted radiofrequency ablation (RFA) and open surgical resection for OO and report a rare case of malignant transformation to osteosarcoma (OS).

**Methods:**

In this retrospective study, we reviewed data from 84 patients with OO treated between January 2014 and January 2024. Twenty-eight patients underwent robot-assisted RFA and 56 underwent open surgical resection. The evaluated outcomes included operative time, intraoperative blood loss, hospitalization duration, pain relief, and total medical costs.

**Results:**

The RFA group demonstrated a clear minimally invasive advantage, with significantly less intraoperative blood loss (6.4 vs. 78.2 ml, p < 0.001) and a shorter hospital stay (3.9 vs. 6.6 days, p = 0.003). However, the procedure required a longer operative time (130.0 vs. 97.6 min, p = 0.003) and incurred higher costs (35,715 vs. 26,959 CNY, p = 0.022). Both groups achieved a 100% initial success rate. The RFA group exhibited superior early pain relief, with significantly lower VAS scores on postoperative day 1 (p < 0.001). Two cases of malignant transformation to OS occurred in the RFA cohort.

**Conclusion:**

Robot-assisted RFA offers minimally invasive advantages over open resection, providing reduced blood loss, shorter hospitalization, and faster pain relief. However, its associated longer operative time, higher cost, and the potential for rare malignant transformation necessitate careful patient selection and stringent long-term follow-up.

## 1 Introduction

Osteoid osteoma (OO) is a benign bone tumor, accounting for approximately 3%–4% of all bone neoplasms and occurring predominantly in children and adolescents (1–3). For patients whose symptoms are refractory to nonsteroidal anti-inflammatory drugs (NSAIDs), the primary interventional options are open surgical resection and computed tomography (CT)-guided radiofrequency ablation (RFA) (4). While open resection has long been the historical mainstay, with success rates ranging from 90% to 100% (5–7), minimally invasive percutaneous RFA has gained widespread acceptance in recent decades due to its reduced tissue trauma and comparably favorable outcomes, boasting success rates of 80% to 100% (8). Recent systematic reviews and meta-analyses have further corroborated that RFA is as safe and effective as traditional surgical excision, offering comparable high success rates while significantly reducing procedural invasiveness and complication rates (9). Building upon this, emerging technologies such as robotic navigation have been introduced to overcome the inherent limitations of conventional CT-guided RFA, with the goal of minimizing radiation exposure and enhancing targeting precision (10). The latest advancement in this domain is robotic assistance, which aims to augment the precision and safety of percutaneous ablation (11).

However, the adoption of RFA must be tempered by considerations of long-term safety. A concerning, albeit rare, complication is malignant transformation. This phenomenon has been observed after incomplete RFA of malignant tumors in the liver and lung (12, 13), raising the question of whether the procedure could induce similar changes in benign lesions. However, a causal link in the context of benign bone tumors like OO remains unclear, with only isolated case reports documenting such an occurrence (14).

To address these considerations, this study had two primary objectives. First, we sought to perform a comparative analysis of the perioperative outcomes—encompassing operative efficiency, blood loss, hospital stay, pain control, and cost—between robot-assisted RFA and open resection. Second, we present a detailed case of OO transforming into osteosarcoma (OS) post-RFA, using this compelling clinical narrative to emphasize the indispensable role of careful patient selection and sustained postoperative surveillance.

## 2 Materials and methods

### 2.1 Study design and patients

This retrospective study enrolled patients diagnosed with OO who underwent surgical treatment between January 2014 and January 2024. To reduce confounding effects, controls were matched to cases at a 2:1 ratio based on age and sex. The final cohort consisted of 84 patients, among whom 28 were treated with robot-assisted RFA and 56 were treated with open surgical resection.

### 2.2 Surgical techniques

#### 2.2.1 Open resection

Tumor localization was guided by preoperative imaging and augmented by intraoperative imaging when necessary. A skin incision was made, followed by thorough curettage of the nidus and the surrounding affected tissue. If required, the residual cavity was filled with autologous or allogeneic bone grafts. Internal fixation was applied in selected cases to prevent postoperative fracture (15, 16).

#### 2.2.2 Robot-assisted RFA

**Figure 1:**
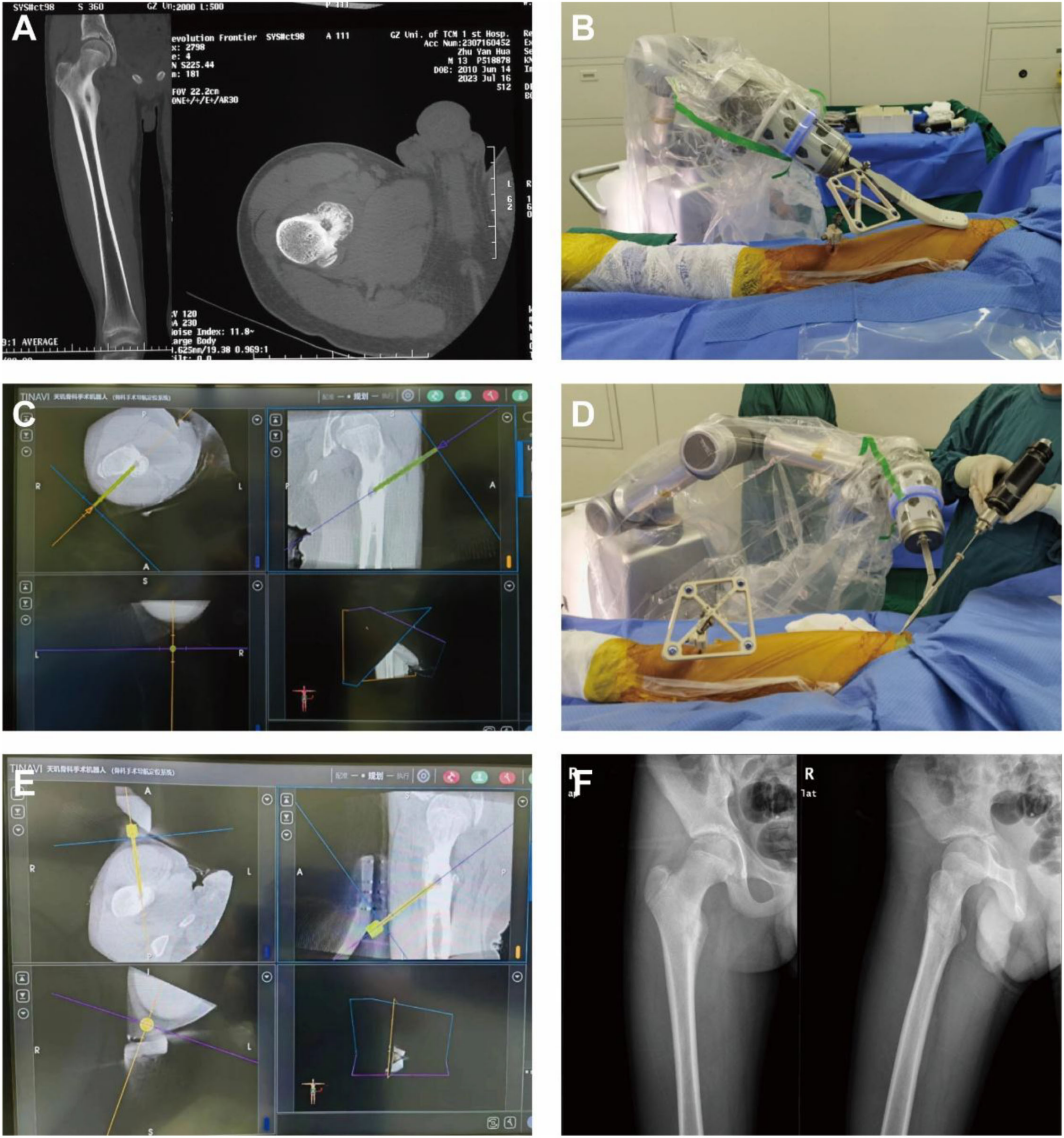
Sequential steps of robot-assisted RFA. The procedure began with **(A)** a preoperative CT scan identifying the OO, followed by **(B)** intraoperative localization and scanning. Subsequently, **(C)** 3D modeling and trajectory planning were performed, guiding **(D)** the insertion of the guide needle. **(E)** Radiofrequency ablation was then applied. **(F)** A follow-up radiograph confirmed the treatment outcome.

Lesion localization was performed using an O-arm X-ray system. The acquired three-dimensional images were reconstructed and uploaded to a robotic navigation system (TINAVI Medical Technologies, China) for preoperative planning. Under robotic or CT guidance, a puncture needle was advanced along the preoperatively planned trajectory. After confirming optimal positioning, a tumor tissue sample was obtained for pathological analysis. RFA was then conducted at 80–90 °C for 3–5 minutes, with temperature adjusted according to lesion size and morphology (8, 11, 17) (**Figure 1**).

### 2.3 Data collection

The collected intra-operative data included operation time and estimated blood loss. Post-operative parameters encompassed the length of hospital stay, total hospitalization costs, pathological findings, and success (categorized as initial and long-term) rates.

The total hospitalization costs were defined as all medical expenses incurred during the patient’s hospital stay. Initial success was defined as complete pain relief within the first seven days after the procedure. Long-term success was defined as sustained pain relief without recurrence or malignant transformation during a follow-up period of at least 24 months.

### 2.4 Statistical analysis

Data were analyzed with SPSS software (version 25, IBM Corp., Chicago, IL, USA). Continuous variables that were normally distributed were presented as means ± standard deviations (SDs) and compared with Student’s t tests; otherwise, they were summarized as median and interquartile range (IQR) and compared with Mann-Whitney U tests. Categorical variables were expressed as frequencies with percentage and compared using chi-square or Fisher’s exact test, whichever was appropriate. A two-sided p value < 0.05 was considered statistically significant.

## 3 Results

### 3.1 Baseline characteristics

The analysis included 84 patients, comprising 28 in the robot-assisted RFA group and 56 in the open resection group. The two groups were well-balanced at baseline (**Table 1**), with no statistically significant differences identified in key demographic and clinical characteristics. Specifically, comparisons of mean age (17.4 vs. 19.4 years, p = 0.498), sex distribution (75% vs. 64% male, p = 0.983), lesion size (p = 0.072), and lesion location (p = 0.236) all yielded non-significant results. Furthermore, the median follow-up duration was comparable between the open resection group (52.5 months; IQR, 38.0–72.5) and the robot-assisted RFA group (46.0 months; IQR, 31.5–60.5) (p = 0.184), ensuring the reliability of subsequent long-term outcome comparisons.

**Table 1:** Comparison of baseline characteristics between the robot-assisted RFA and open resection groups.

Variables	Open surgery (n = 56)	Robot-assisted RFA (n = 28)	P value
Gender (n, %)			0.983
Male	42 (75.0%)	18 (64.3%)	
Female	14 (25.0%)	10 (35.7%)	
Age (years, mean ± SD)	17.4 ± 11.8	19.4 ± 14.7	0.498
Lesion size (mm, mean ± SD)	12.1 ± 9.1	8.8 ± 4.0	0.072
Lesion location (n, %)			0.236
Femur	35 (62.5%)	21 (75.0%)	
Tibia	11 (19.6%)	3 (10.7%)	
Humerus	4 (7.1%)	1 (3.6%)	
Fibula	1 (1.8%)	0 (0.0%)	
Other	5 (8.9%)	3 (10.7%)	
VAS before surgery	6.44	6.52	0.591
Follow-up duration (months, median [IQR])	52.5 (38.0–72.5)	46.0 (31.5–60.5)	0.184

RFA, radiofrequency ablation; SD, standard deviation; VAS, visual analog scale; IQR, interquartile range.

### 3.2 Perioperative outcomes

Compared with the open resection group, the RFA group demonstrated significantly reduced intraoperative blood loss (6.4 vs. 78.2 ml, p < 0.001) and a shorter duration of hospitalization (3.9 vs. 6.6 days, p = 0.003). However, these benefits were achieved at the cost of a longer operative time (130.0 vs. 97.6 min, p = 0.003) and higher medical costs (35,715 vs. 26,959 CNY, p = 0.022) (**Table 2**). These findings highlight the minimally invasive profile of RFA, which is counterbalanced by increased procedural time and financial expense.

**Table 2:** Comparative analysis of key perioperative outcomes between robot-assisted RFA and open resection groups.

Evaluated outcomes	Open surgery (n = 56)	Robot-assisted RFA (n = 28)	P value
Operation time (minutes, mean ± SD)	97.6 ± 41.5	130.0 ± 45.7	0.003
Blood loss (ml, mean ± SD)	78.2 ± 42.2	6.4 ± 9.3	< 0.001
Times of intra-operative scans (mean ± SD)	0.6 ± 0.6	2.8 ± 0.8	0.001
Hospitalization stays (days, mean ± SD)	6.6 ± 6.4	3.9 ± 2.0	0.003
Pathological examination findings (n, %)			1.000
Positive	40 (71.4%)	20 (71.4%)	
Negative	16 (28.6%)	8 (28.6%)	
Initial success rate	100% (56/56)	100% (28/28)	1.000
Long-term success rate ^a^	100% (56/56)	92.9% (26/28)	0.326
Medical costs (CNY, mean ± SD)	26959.69 ± 20203.56	35715.62 ± 14956.96	0.022

RFA, radiofrequency ablation; SD, standard deviation, CNY, Chinese Yuan.

^a^ The two cases of long-term failure in the robot-assisted RFA group represent the patients who experienced local recurrence accompanied by malignant transformation.

### 3.3 Efficacy and pain assessment

**Figure 2:**
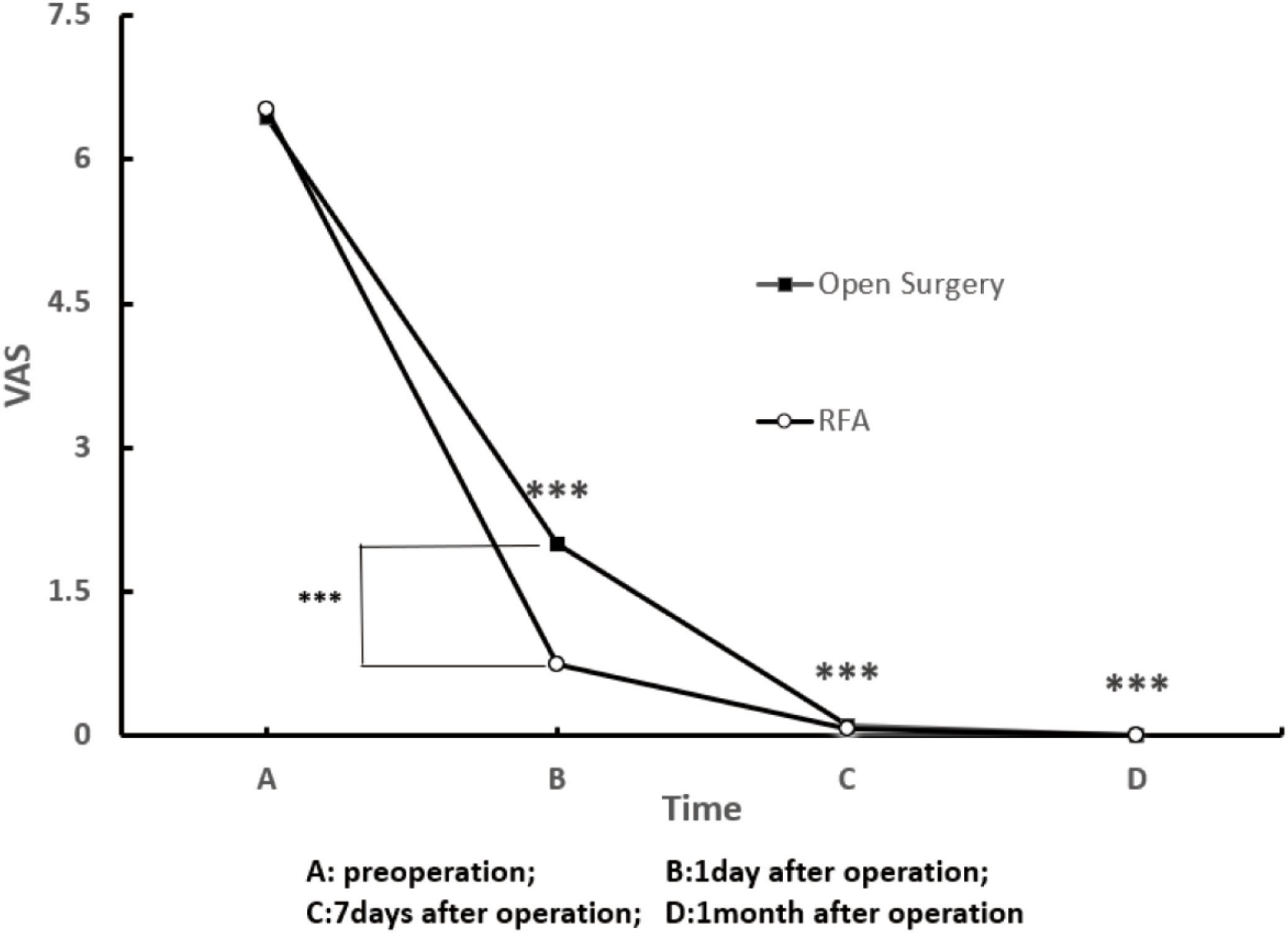
Dynamic changes in VAS scores following surgery. Comparison of pain scores at baseline (preoperative), postoperative day 1, day 7, and month 1 between the two treatment groups. ***p < 0.001, compared with the preoperative value within the same group.

The initial success rate, defined as pain relief within seven days, was 100% in both groups. The long-term success rate was 100% (56/56) in the open resection group and 92.9% (26/28) in the RFA group (p = 0.326). Notably, the two cases of long-term clinical failure in the RFA cohort were precisely the patients who experienced local recurrence accompanied by subsequent malignant transformation, which are detailed in the following case report. This was corroborated by the longitudinal assessment of Visual Analog Scale (VAS) scores (**Figure 2**). Both groups exhibited significant reductions in pain at postoperative day 1 and 7 compared to baseline (all p < 0.001). Notably, the RFA group demonstrated superior pain control at the early postoperative stage, as evidenced by significantly lower VAS scores than the open resection group on day 1 (0.75 vs. 2.00, p < 0.001). The preoperative and day 7 VAS scores were comparable between the two groups (Preoperative: 6.52 vs. 6.44; Day 7: 0.07 vs. 0.11). The rate of positive pathological confirmation was identical between groups (62.5%).

### 3.4 Case report

Two patients in the RFA group experienced recurrence accompanied by malignant transformation. A representative case is described below.

**Figure 3:**
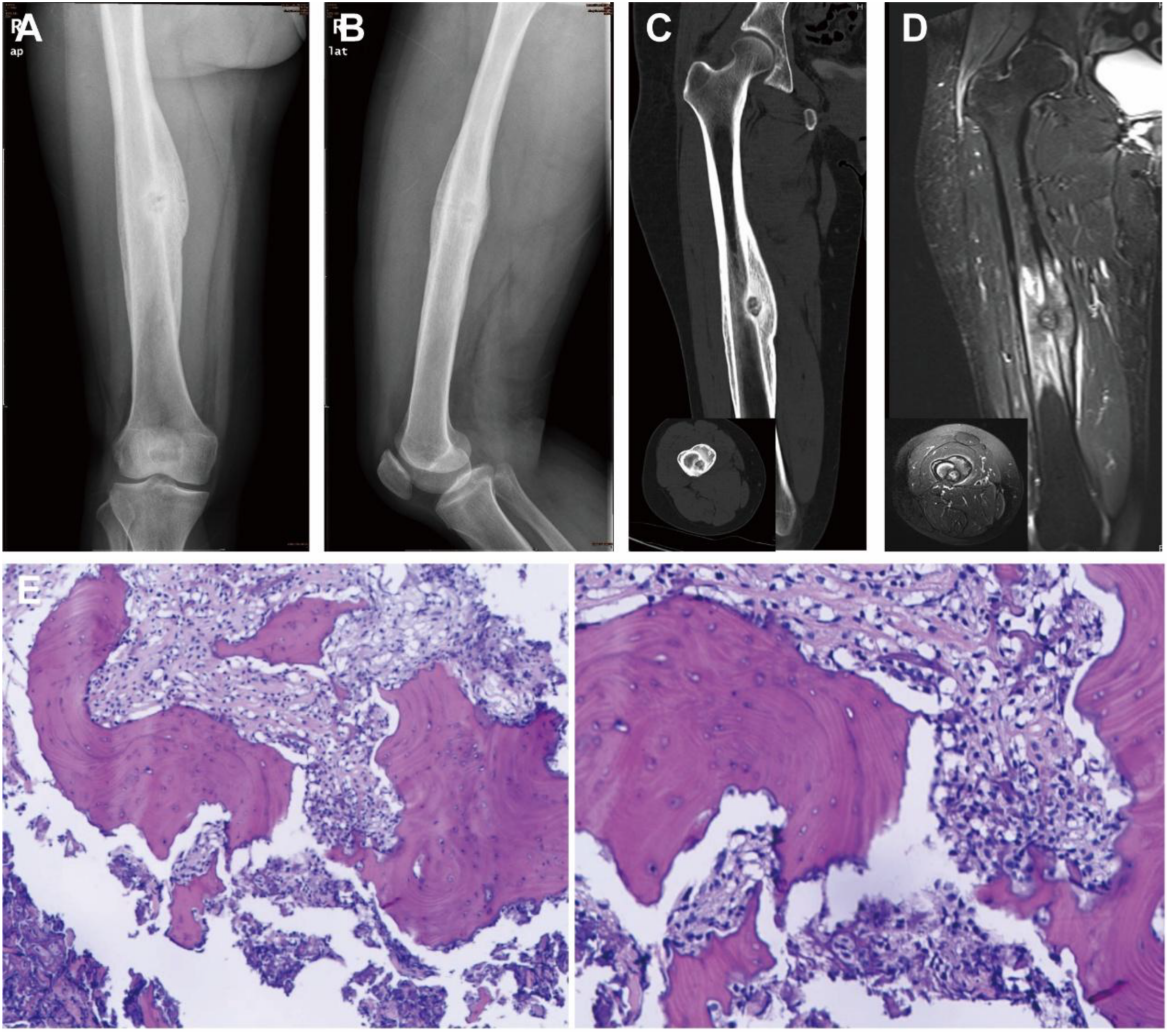
Diagnostic workup for a 55-year-old female with an OO. **(A-D)** Preoperative imaging studies consistent with OO. **(E)** Postoperative histopathological analysis confirming the diagnosis of OO without malignant features.

**Figure 4:**
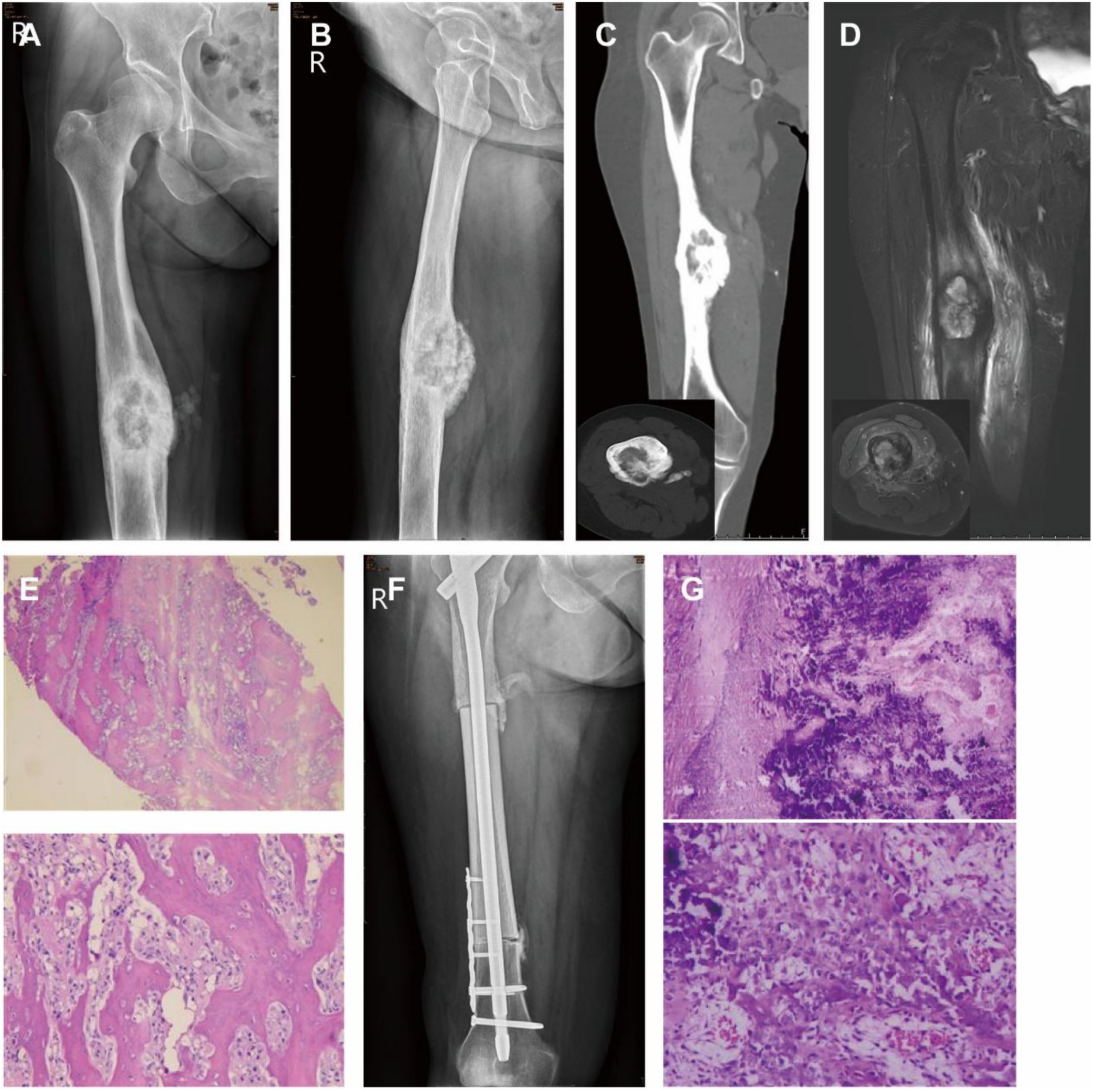
Malignant transformation to OS and subsequent management. **(A-D)** Surveillance imaging revealed local recurrence. **(E)** Core needle biopsy of the recurrent lesion was suggestive of invasive osteoblastoma; however, multidisciplinary review established the final diagnosis of OS. **(F)** The patient was managed with neoadjuvant chemotherapy, followed by wide tumor resection, allogeneic bone grafting, intramedullary nail fixation. **(G)** Definitive histopathological analysis of the resected specimen confirmed the diagnosis of OS.

A 55-year-old woman presented with a one-month history of right femoral pain. CT findings were consistent with an OO of the right femur. The patient subsequently underwent robot-assisted RFA. Postoperative histological analysis confirmed the diagnosis of OO, with no evidence of malignancy (**Figure 3**). Surveillance CT at 7 months postoperatively showed no signs of recurrence. However, a follow-up CT performed 16 months later demonstrated local recurrence. A core needle biopsy of the recurrent lesion was suggestive of invasive osteoblastoma. Upon multidisciplinary review by two pathologists and two radiologists, the final diagnosis was amended to OS. The patient was subsequently managed according to standard OS protocols (18, 19), which included neoadjuvant chemotherapy, wide tumor resection coupled with allogeneic bone grafting and intramedullary nail fixation. Definitive pathological examination of this resection specimen confirmed the diagnosis of osteosarcoma. The patient then completed the protocol with adjuvant chemotherapy. (**Figure 4**).

This rare case highlights the critical importance of long-term follow-up after RFA, as it underscores the potential, albeit uncommon, for malignant transformation.

## 4 Discussion

This study demonstrates that robot-assisted RFA is a viable alternative to open resection for OO, achieving equivalent initial success while offering distinct perioperative benefits. Specifically, the RFA approach resulted in markedly less blood loss, a shorter hospitalization, and more rapid early pain control. However, these advantages were accompanied by longer operative times and higher direct medical costs. A notable finding was the identification of two cases of malignant transformation in the RFA group, detailed in a representative case report, which highlights a critical need for sustained postoperative surveillance.

The observed reductions in surgical trauma and recovery time are consistent with the established benefits of percutaneous ablation techniques (**Table 3**). Previous work by Meng et al. (20) has documented similar advantages for CT-guided RFA over open surgery, including shorter hospital stays and reduced perioperative morbidity. Our data extend this literature by indicating that the integration of a robotic navigation system can enhance procedural precision, a feature particularly valuable for lesions in high-risk locations. The longer operative duration observed with robotics is likely attributable to the workflow requirements of preoperative planning and system setup, which may improve with standardized protocols and growing surgical familiarity.

**Table 3:** Literature review of selected studies on open resection and radiofrequency ablation for osteoid osteoma ^*^.

Authors	No. of Patients	Type of surgery	Length of stay (days)	Blood loss (ml)	Recurrence or malignant transformation
Etemadifar et al. (6)	19	Open surgery	na	na	0
Mallepally et al. (7)	5	Open surgery	na	na	0
Meng et al. (20)	53	Open surgery	11.37	133.43	1
Yu et al. (32)	16	Open surgery	6	125	0
This study	56	Open surgery	6.6	78.2	0
Wang et al. (11)	62	RFA	na	na	0
Meng et al. (20)	15	RFA	2.2	6.87	0
Faddoul et al. (22)	8	RFA	na	na	1
Yu et al. (32)	12	RFA	1	1	1
Shields et al. (33)	42	RFA	na	na	7
This study	28	RFA	3.9	6.4	2

No., number; RFA, radiofrequency ablation; na, not applicable.

^*^Superscript numbers correspond to the reference list.

RFA is widely recognized as an effective modality for pain control in OO, a consensus supported by both previous literature and the present findings. Albisinni et al. (21) documented complete pain resolution (VAS = 0) within one month post-RFA in their cohort, while Faddoul et al. (22) reported a sharp decline in mean VAS scores from 7.55 preoperatively to 2.56 on postoperative day 1. Corroborating these reports, our data demonstrated a steeper early pain reduction curve with RFA compared to open resection, evidenced by significantly lower VAS scores at 24 hours. This rapid analgesia holds direct clinical relevance, potentially facilitating earlier mobilization and shortening the rehabilitation pathway, particularly in pediatric and adolescent populations. By one week, however, pain resolution was nearly complete in both groups, reaffirming the high efficacy of both techniques for definitive symptomatic control.

A critical consideration in employing RFA, however, is its associated risk of recurrence and the rare but serious potential for malignant transformation. The literature has consistently linked recurrence to lesion morphology, with larger size and an eccentricity index (EI) ≥ 3 serving as reported risk factors (23, 24). The geometrical mismatch between the spherical ablation zone and elongated or irregular nidi can lead to incomplete ablation, which remains a principal technical limitation of RFA (25). Our study adds a further dimension to this risk profile: we observed two cases of malignant transformation to OS in the RFA cohort (2/28), a complication not encountered in the open resection group. The representative case involved a sizable lesion, implying that lesion dimensions may influence not only recurrence risk but also the propensity for malignant degeneration following RFA. Consequently, the presence of high-risk morphological features on preoperative imaging should prompt careful deliberation between minimally invasive benefits and long-term oncological safety, warranting either alternative surgical strategies or a structured, long-term surveillance regimen.

The malignant transformation observed, though extraordinarily rare, carries profound clinical implications. This phenomenon, while reported following sublethal RFA in malignancies like hepatocellular carcinoma (12, 13, 26), is virtually undocumented in the natural history of OO, a tumor known for its benign course (1). The documented progression of OO to osteoblastoma is itself uncommon (27), and the subsequent transformation of osteoblastoma to OS, as in the mandibular case reported by Woźniak et al. (28), is rarer still. The trajectory from OO to OS in our patient posits that incomplete ablation or a distinct tumor biology may, in rare instances, enable such aggressive progression. While causality remains unproven, this finding mandates scrupulous lesion targeting, adequate ablation margins, and a structured long-term follow-up regimen, reinforcing that even benign lesions treated with ablation require vigilant monitoring.

The significant cost premium of robot-assisted RFA, driven by technology and procedural demands, may curtail its accessibility in resource-constrained environments. A formal cost-benefit analysis, especially in morbidity-sensitive pediatric cohorts, is therefore essential to define its appropriate role in the therapeutic arsenal. Although the direct medical costs were significantly higher in the robot-assisted RFA cohort—primarily driven by the capital investment in robotic equipment—this initial financial burden may be partially offset by substantial indirect economic and social benefits. The significantly shorter hospital stay (3.9 vs. 6.6 days) not only translates to reduced bed occupancy costs but also potentially lowers the risk of hospital-acquired infections. Furthermore, the rapid early pain relief facilitates faster rehabilitation. Considering our predominantly young patient population, this accelerated recovery is particularly valuable; it allows patients to return to school earlier and enables their parents or caregivers to resume work sooner, thereby minimizing lost productivity. Therefore, when viewed through a broader socio-economic lens, the overall cost-effectiveness of robot-assisted RFA might be considerably more favorable than what direct medical expenses alone suggest.

Our study’s conclusions are tempered by several limitations. First, due to the retrospective and single-center nature of the study, the sample size remains relatively modest. While increasing the sample size is challenging given the rarity of OO, future multi-center studies are needed to increase the statistical power and reliability of these findings. Second, although we established a minimum 24-month follow-up period to evaluate long-term outcomes, retrospective data collection inevitably introduces variability in surveillance duration. As starkly evidenced by the local recurrence and malignant transformation at 16 months in our cohort, extended longitudinal surveillance is therefore mandatory for a definitive assessment of true recurrence rates (29–31).

## 5 Conclusion

To conclude, robot-assisted RFA is a highly effective, minimally invasive alternative that accelerates early recovery and pain relief compared to open resection. As a critical take-away message for clinical practice, the higher direct costs and longer procedural times must be carefully weighed, and most importantly, the exceedingly rare but severe risk of malignant transformation mandates that RFA should only be performed when rigorous patient compliance with long-term surveillance can be guaranteed.

## Data Availability

The raw data supporting the conclusions of this article will be made available by the authors, without undue reservation.
